# The regulatory mechanisms of active ingredients in traditional Chinese medicine for inflammation in Parkinson disease: A review

**DOI:** 10.1097/MD.0000000000044392

**Published:** 2025-10-17

**Authors:** Yuxuan He, Jingyi Wang, Chunmiao Ying, Weixiao Liang, Zaitian Yin, Jing Gao, Boqiao Wang, Xiaohui Zhao, Yunke Zhang

**Affiliations:** a Neurology Department, The First Affiliated Hospital of Henan University of Traditional Chinese Medicine, Zhengzhou, Henan, China; b University of California, Irvine, CA; c Henan University of Traditional Chinese Medicine, Zhengzhou, Henan, China.

**Keywords:** herbal medicine, inflammatory factors, inflammatory pathways, microglia, neuroinflammation, Parkinson disease

## Abstract

Parkinson disease (PD) is the second most common neurodegenerative disorder worldwide, affecting countless patients. Inflammation is a major pathogenic factor in PD. The regulation of inflammation involves multiple processes, including glial cell activation, oxidative stress responses, gut–brain axis signaling, and associated molecular pathways. In recent years, some progress has been made in the study of anti-inflammatory therapies for PD; however, their therapeutic efficacy remains limited. Thus, exploring alternative pharmacological interventions may represent a promising direction for managing inflammation in PD. Traditional Chinese medicine (TCM) has been found to exert therapeutic effects on PD-related inflammation, suggesting that further development of TCM could be beneficial. Therefore, focusing on the discovery and development of TCM-based treatments is worth pursuing. This review summarizes the major active components that show efficacy against PD inflammation and outlines their underlying mechanisms, with the aim of providing new perspectives for identifying alternative therapeutic strategies.

## 1. Introduction

In the year 1817, James Parkinson embarked on a pioneering journey into the realm of neurology by meticulously chronicling the condition of 6 individuals. His observations unveiled the quintessential features of what would later bear his name: Parkinson disease (PD). These included involuntary tremors, weakened muscle strength, limited mobility, and a noticeable forward stoop, even when supported.^[1]^ Fast forward to the present, PD emerges as the globe’s second most common neurodegenerative ailment. The standardized prevalence rate is about 8 to 18/100,000 person-years, with a prevalence of 1% in the elderly population over the age of 60 and a positive correlation between prevalence and age. According to statistics, only in 2016, the number of people affected by PD reached 6 million people worldwide, plaguing countless patients.^[2–4]^

At its core, PD is marked by the progressive degeneration and loss of dopaminergic neurons within the SNpc, leading to diminished levels of dopamine in the striatum and the formation of LBs, chiefly composed of α-synuclein.^[5]^ Characterized by its stealthy onset and slow progression, PD manifests through resting tremors, bradykinesia, and muscular stiffness.^[6]^ A significant contributor to the disease’s progression is excessive neuroinflammation, which jeopardizes dopaminergic neurons.^[7]^ Despite Levodopa status as the most efficacious clinical remedy,^[8,9]^ its side effects and the potential for resistance drive the quest for alternative treatments. Herein lies the promise of TCM – with its rich theoretical heritage and extensive history in treating PD, herbal medicine stands as a hope for novel interventions. This discourse delves into the prowess of TCM in mitigating PD-associated inflammation.

## 2. PD and inflammation

Inflammation in PD encompasses both neuroinflammation and peripheral inflammation. Inflammation is the body’s defense against external damage, destroying pathogens and repairing damage. Yet, this protective mechanism can spiral out of control, becoming detrimental to the CNS and leading to the deterioration of dopaminergic neurons within the SNpc. The catalysts for this inflammatory cascade – ROS and RNS – are byproducts of oxidative stress, exacerbating neuroinflammation and neuronal damage. Peripheral inflammation, traversing the gut-brain axis, can penetrate the BBB, igniting neuroinflammation and compromising dopaminergic neurons in the SNpc.

Central to the PD narrative is α-synuclein, a neuroprotein ubiquitously expressed in various brain regions (SN, midbrain, etc) and peripheral sites like the intestines, playing a pivotal role in PD-related inflammation.^[10]^ Structurally, α-synuclein comprises 3 domains: an N-terminal α-helical region that interfaces with lipid vesicles and cell membranes, a hydrophobic NAC domain, and a C-terminal replete with negative charges and hydrophilicity.^[11]^ Ordinarily, α-synuclein remains highly soluble and aggregation-resistant. Pathological shifts in its structural domains, however, can trigger misfolding into insoluble oligomers and LBs, disrupting mitochondrial integrity and synaptic functionality and causing the apoptosis and necrosis of dopaminergic neurons.^[12]^ The interplay of microglia-induced neuroinflammation and peripheral inflammation is instrumental in the onset of PD, highlighting the intricate dynamics at play in this neurodegenerative disease.^[13]^

### 2.1. Neuroinflammation in PD

In 1988, McGeer et al identified significant microglial activation in the SNpc of a postmortem PD patient’s brain,^[14]^ highlighting neuroinflammation’s role in PD pathogenesis. Emerging evidence has solidified that glial cells, including microglia and astrocytes, are pivotal in PD-associated neuroinflammation.^[15–19]^ Within the CNS, glial cells display either a resting or activated state.^[20]^ Typically, glial cells remain inactive in a healthy physiological state, with neurons and astrocytes working together to suppress microglial activation.^[21]^ Microglia play a vital role in maintaining cerebral homeostasis through dynamic surveillance, synaptic pruning, and support for neural circuit formation.^[22,23]^ Astrocytes provide neuronal support, contribute to BBB integrity, and offer nutritional, metabolic, synaptic modulation, neurotransmitter reuptake, and regulation of K^+^ and water balance.^[24,25]^ Glial cells utilize surface PRRs to detect PAMPs and DAMPs, initiating the clearance of harmful stimuli.^[26]^ Notably, α-synuclein aggregation and cytokines trigger glial cell activation via TLRs,^[27]^ removing damaging agents.^[28,29]^ However, excessive glial activation can provoke widespread inflammation and leukocyte infiltration, damaging dopaminergic neurons in chronic neuroinflammation.^[30]^ Interestingly, α-synuclein polymorphisms may induce varied microglial activation states and functionalities.^[31]^

**Figure 1.**
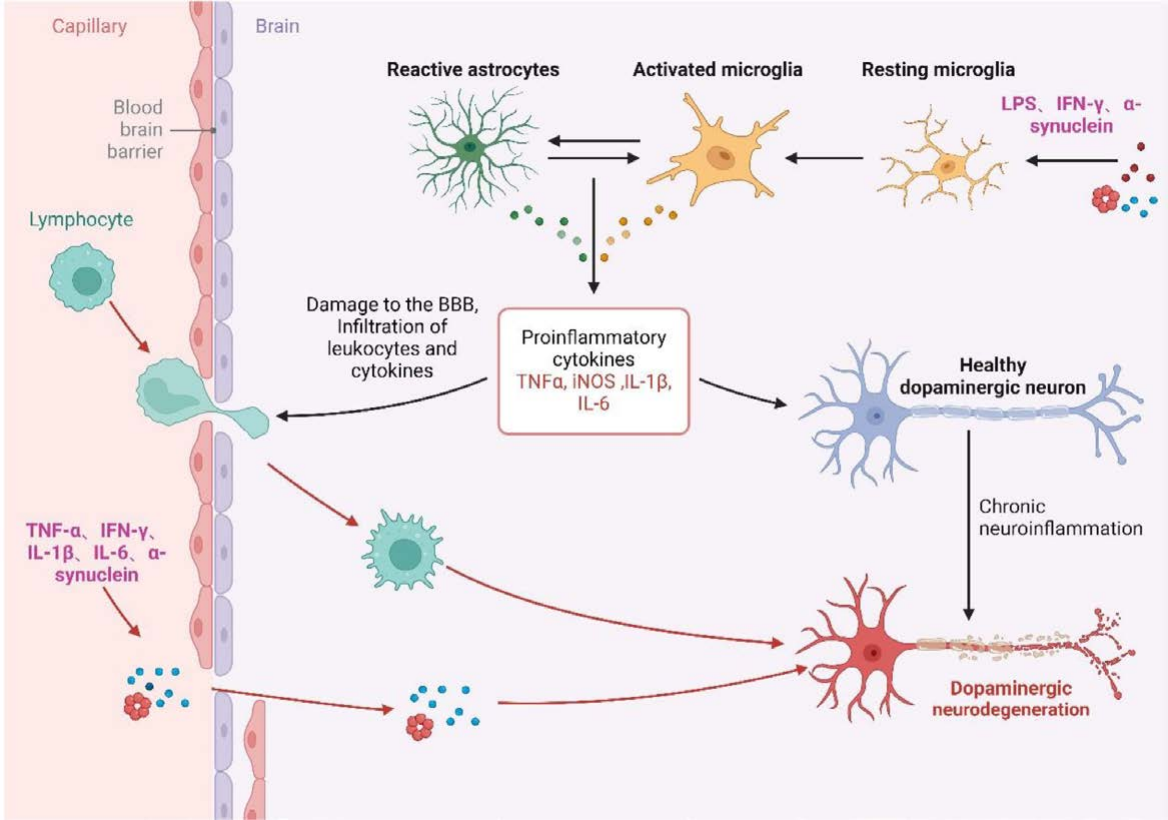
Inflammation in PD. Inflammation in PD encompasses both neuroinflammation and peripheral inflammation. Activated microglia and astrocytes can induce neuroinflammation by releasing IL-1β, IL-6, TNF-α, and iNOS. Peripheral inflammation, on the other hand, is caused by alpha-synuclein and T cells producing IL-1β, IL-6, TNF-α, and IFN-γ. These inflammatory pathways continuously attack dopaminergic neurons, leading to neurodegeneration. IFN-γ = interferon-γ, PD = Parkinson disease.

Activated microglia, characterized by their ameboid shape, can differentiate into M1 (pro-inflammatory) and M2 (anti-inflammatory) phenotypes.^[32]^ M1 microglia, identifiable by markers such as Iba-1^[33]^ and CD11b,^[34]^ are induced by cytokines like lipopolysaccharide (LPS) and IFN-γ^[35]^ to activate inflammatory pathways (TLR4/NF-κB, p38/MAPK, JAK2/STAT3^[36]^) and upregulate pro-inflammatory cytokines (IFN-γ, tumor necrosis factor-alpha [TNF-α], interleukin-1β [IL-1β], interleukin-6 [IL-6], IL-18^[37,38]^), as illustrated in Figure 1. Besides, M1 microglia produce vast amounts of ROS, HO-1, inducible nitric oxide synthase (iNOS), and cyclooxygenase-2 (COX-2),^[39]^ contributing to inflammation and pathogen clearance.^[38]^ Conversely, M2 microglia, induced by IL-4, and IL-13,^[40]^ Antagonizing M1 microglia, release IL-10, TGF-β, and neurotrophic factors, neuroprotection and inflammation mitigation.^[41]^

Astrocytes can differentiate into A1 (pro-inflammatory) and A2 (anti-inflammatory) phenotypes,^[42]^ with GFAP^[43]^ serving as a marker of their activation. A1 astrocytes are induced by cytokines such as LPS, IL-1β, TNF-α, and IFN-γ.^[44,45]^ These cytokines activate inflammatory pathways (TLR/NF-κB, JAK/STAT^[45,46]^) and upregulate inflammatory cytokines (IL-1β, IL-6, TNF-α, NO^[45,47,48]^), promoting neuroinflammation. Conversely, the A2 phenotype, activated by TGF-β, can inhibit inflammation and facilitate neuronal repair.^[48]^

In addition, adaptive immunity is involved in neuroinflammation in PD. CD3,^[14]^ CD4,^[49,50]^ and CD8^[51]^ T cells can trigger inflammation by infiltrating the CNS, and in 1988, McGeer et al found that CD3 T cells infiltrated the brains of PD patients.^[14]^ Since then, CD4, CD8 T cells were found in SNpc. B cells are also involved in the pathogenesis of PD, but are poorly understood and are still in the exploratory stage.^[52,53]^ The BBB consists of vascular endothelial cells, basement membrane layer and astrocytes, which isolate the CNS from the peripheral circulation, preventing harmful substances from entering the brain.^[54]^ Neuroinflammation in PD is often accompanied by disruption of the BBB when peripheral CD4^+^ T cells then enter the CNS and cause inflammation. Th1, Th2, and Th17 are all involved in the pathogenesis of PD. IFN-γ and TNF-α released by Th1 can activate microglia and cause neuroinflammation.^[55]^ In contrast, IL-4, IL-5, IL-10, and IL-13, released by Th2 cells, can inhibit neuroinflammation,^[56]^ and Th17 can produce IL-17 and other inflammatory factors to promote inflammation.^[57]^ Thus, modulation of T cells is also able to reduce neuroinflammation.

#### 2.1.1. TLR4/NF-κB pathway

**Figure 2.**
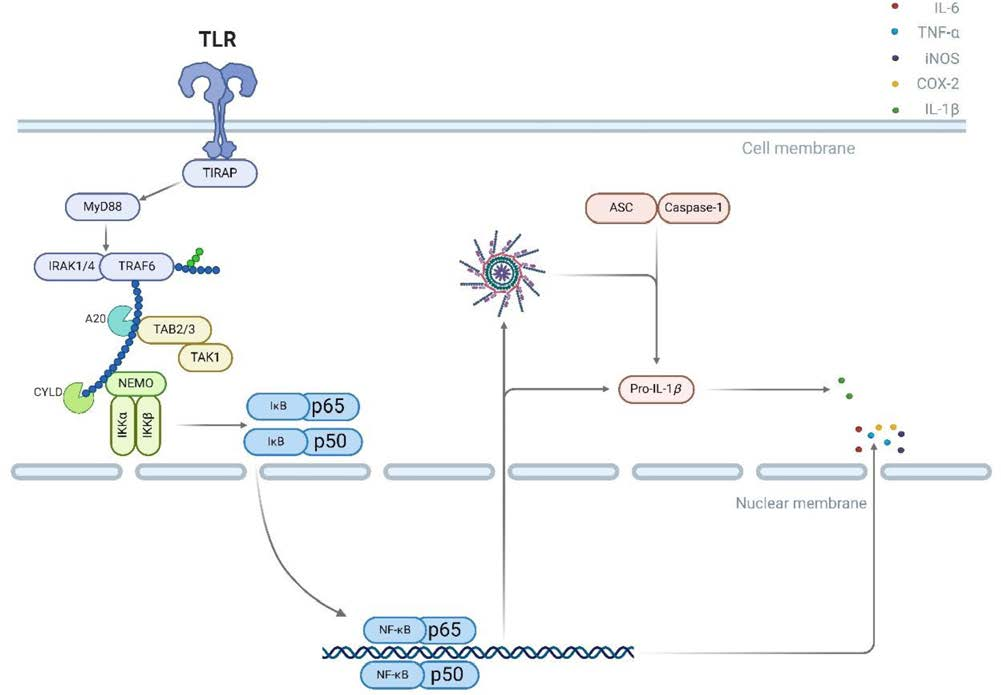
TLR4/NF-κB pathway. After TLR4 is activated, the signal is transduced to IκB through MYD88, induction of phosphorylation of IκBα/IκBβ proteins, entry of p50 and p65 into the nucleus, transcription of pro-inflammatory genes, and upregulate inflammatory factors TNF-α, IL-6, COX-2, iNOS. COX-2 = cyclooxygenase-2, iNOS = inducible nitric oxide synthase.

The TLR4/NF-κB pathway, activated by external stimuli like α-synuclein and cytokines,^[28,29,35]^ triggers a signaling cascade involving TIRAP and MYD88, leading to the activation of TAK1, IKKs, induction of phosphorylation of IκBα/IκBβ proteins, entry of p50 and p65 into the nucleus,^[58]^ transcription of pro-inflammatory genes, and upregulate inflammatory factors TNF-α, IL-6, COX-2, iNOS^[59,60]^ and other inflammatory factors. Then Active, the NF-κB/NLRP3 signaling pathway. The NF-κB/NLRP3 pathway is initiated by microglial activation. Microglia first activate NF-κB through cytokine-activated TLR to the assembly of a complex and release Pro-Caspase-1, Pro-IIL-1β, Subsequent hydrolysis of ProCaspase-1 to Caspase-1, that activates caspase-1, and the concomitant recruitment of ASCs, cleaves Pro-IL-1β to IL-1β resulting in the secretion of IL-1β,^[61,62]^ as illustrated in Figure 2.

#### 2.1.2. p38/MAPK pathway

**Figure 3.**
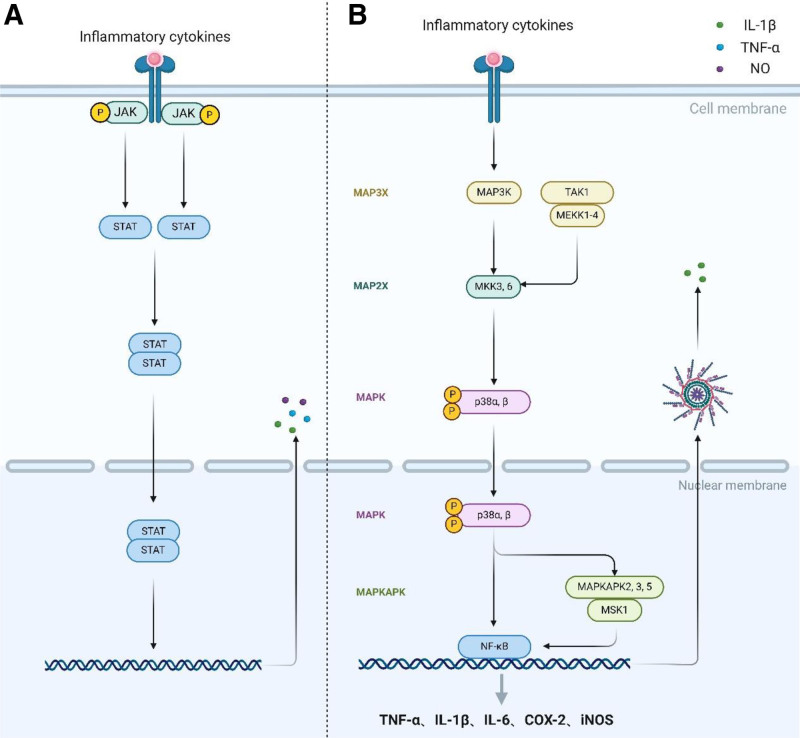
p38/MAPK and JAK2/STAT3 pathway. As illustrated in (B), the activation of the classical MAPK pathway unfolds across 3 tiers: MAP3K, MAP2K, and MAPK. Phosphorylated p38α/β translocates to the nucleus and activates the NF-κB pathway, leading to inflammation by releasing inflammatory cytokines such as IL-1β, COX-2, IL-6, and iNOS. As illustrated in (A), phosphorylation of 2 closely spaced JAK2 each activates a STAT3, which then dimerizes and enters the nucleus to bind DNA to activate and transcribe 76 to 78 the release of the inflammatory factors NO, TNF-α, and IL-1β. COX-2 = cyclooxygenase-2, iNOS = inducible nitric oxide synthase.

MAPK pathway is an essential signaling mechanism that relays signals from the cell surface to the nucleus, comprising various subfamilies: ERK, JNK, and p38. The p38 and JNK subfamilies are notably linked to inflammation. The activation of the classical MAPK pathway unfolds across 3 tiers: MAP3K, MAP2K, and MAPK,^[63]^ as illustrated in Figure 3B.

Four isoforms of the p38 MAPK family have been delineated: p38α, p38β, p38γ, and p38δ. p38α and p38β have 75% amino acid sequence homology and are abundantly expressed in neuronal cells and macrophages.^[64,65]^ Recognition of extracellular signals by PRRs triggers signal transduction through TIRAP and MYD88 to TRAF6, orchestrating the regulation of MAP3Ks (e.g., MEKK1-4, TAK1) and MAP2Ks (e.g., MKK3, MKK6),^[66]^ culminating in the phosphorylation of p38. This phosphorylated p38 translocates to the nucleus, activating MSK1/2 and MK2, which in turn can activate the NF-κB pathway,^[67,68]^ leading to inflammation through the phosphorylation of IκB and liberation of p65 from the cytosol. Phosphorylated p65 subunit enters the nucleus, triggering the release of inflammatory cytokines like IL-1β, COX-2, IL-6, and iNOS.^[69,70]^ MK2 modulates the phosphorylation of AU-binding proteins, affecting TNF-α secretion.^[71,72]^ Additionally, the p38 MAPK pathway, by activating the NLRP3 inflammasome, facilitates IL-1β release and induces neuroinflammation.^[67,73]^ Inhibiting the p38 MAPK pathway can diminish NF-κB and NLRP3 expression, thus mitigating neuroinflammation.

#### 2.1.3. JAK2/STAT3 pathway

The JAK/STAT pathway facilitates a cascade of cellular protein interactions, transmitting extracellular chemical signals to the nucleus and activating genes via transcription.^[74]^ This pathway involves ligand-receptor complexes, JAK, which consists of 4 members, and STAT, which consists of 7 members.^[75]^ In the context of a PD rat model induced by α-synuclein, the JAK/STAT pathway mitigates the activation of microglia and reduces the infiltration of macrophages and CD4 T cells into dopaminergic neurons.^[76]^ Inflammatory cytokines, notably IFN-γ and IL-6, act as principal activators of the JAK/STAT pathway,^[76,77]^ prompting ligand-receptor complex dimerization, which in turn activates JAK2. This activation leads to the phosphorylation of JAK2, with 2 proximate JAK2 molecules each activating a STAT3.^[78]^ These dimerized STAT3 molecules then translocate into the nucleus to bind DNA,^[79,80]^ activating and transcribing genes for inflammatory cytokines such as NO, TNF-α, and IL-1β,^[77]^ as illustrated in Figure 3A. Inhibition of the JAK2/STAT3 pathway serves to dampen neuroinflammation.

### 2.2. Oxidative stress and inflammation: a nexus in PD

Oxidative stress, a pivotal element in the cascade leading to inflammation within PD, gained prominence through Langston seminal 1983 discovery.^[81]^ The inadvertent consumption of MPTP was found to impair mitochondrial complex functions, spotlighting oxidative stress as a fundamental catalyst for PD. This breakthrough has enshrined oxidative stress as a cornerstone in PD research. ROS – comprising oxygen free radicals and peroxides with a propensity for free radical formation – stand at the crossroads of the body’s oxidative and antioxidative dynamics, maintaining a delicate redox equilibrium.^[82]^ An excess of ROS in cellular realms precipitates oxidative stress in dopaminergic neurons, rendering them more susceptible to the deleterious mediators secreted by activated microglia.^[83]^ Thus, fine-tuning oxidative stress responses emerges as a strategic lever to curb neuroinflammation.

#### 2.2.1. The mitochondrial matrix: a crucible of oxidative stress

Mitochondria, cellular powerhouses, orchestrate energy production through ATP synthesis via oxidative phosphorylation, whilst concurrently being prolific ROS generators. Compromised mitochondrial efficacy precipitates oxidative stress, undermining dopaminergic neuronal integrity.^[84]^ The mitochondrial architecture, with its outer membrane, intermembrane space, inner membrane, and matrix, houses the electron transport chain within the inner membrane. This assembly, comprised of complexes I-IV, coenzyme Q, and cytochrome C, is the linchpin of oxidative phosphorylation.^[85]^ Under physiological guise, mitochondria engage NADH and FADH2 in a dance with complexes I and II, birthing protons and electrons. These protons traverse into the intermembrane space via complexes I, III, and IV, engendering an electrical potential and proton gradient that catalyzes ATP synthesis from ADP and a phosphate moiety.^[85,86]^

The aberrant agglomeration of α-synuclein inflicts dysfunction upon the electron transport chain in dopaminergic neurons,^[87]^ culminating in the overproduction of superoxide anions (O2^–^). These anions, upon mingling with superoxide dismutase (SOD), give rise to hydrogen peroxide (H2O2), which, in cahoots with metal ions like Fe2^+^, begets hydroxyl radicals (OH^-^).^[88,89]^ This cascade of ROS ignites oxidative stress in dopaminergic neurons. The tumult wrought by mitochondrial malfunctions unleashes ROS and RNS (e.g., NO, iNOS), heralding the microglial activation and the onset of neuroinflammation.^[83,90]^ The Nrf2, a redox-sensitive transcriptional guardian, orchestrates cellular resilience against oxidative stress and inflammation by championing the Nrf2/HO-1 pathway.^[91]^

#### 2.2.2. Nrf2/HO-1 pathway

**Figure 4.**
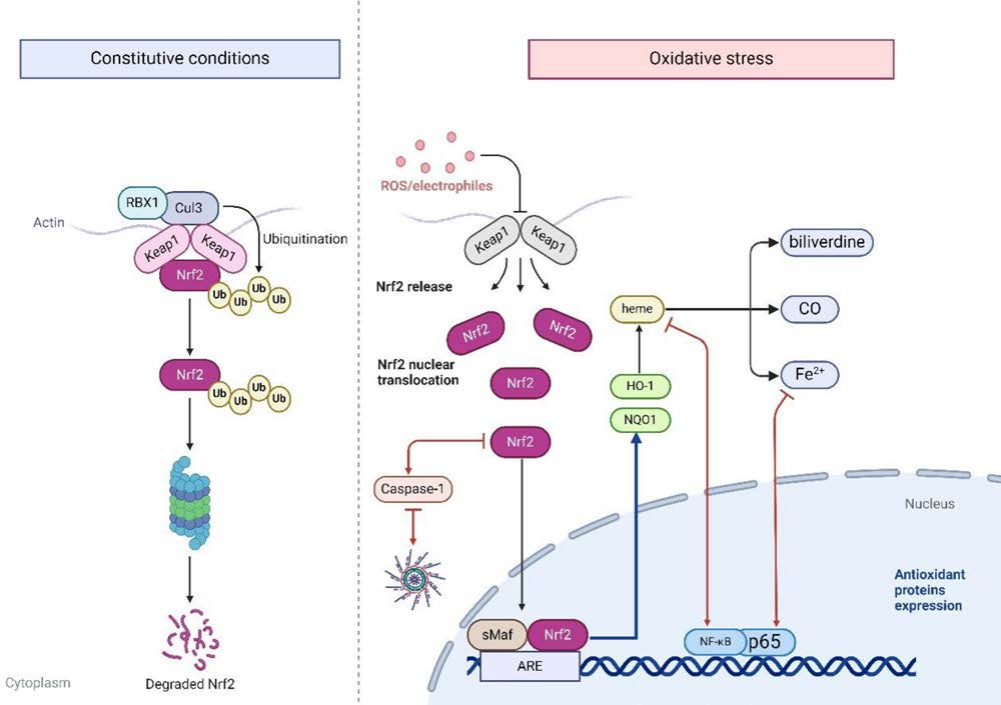
Nrf2/HO-1 Pathway. In the quiescent state, Keap1 binds to Nrf2, curbing its activity through ubiquitination and proteasomal degradation. During oxidative stress, Keap1 segregates from Nrf2, which binds to sMaf and enters the nucleus, transcribing NQO1 and HO-1. HO-1 decomposes heme to produce free Fe ions, carbon monoxide, and biliverdin, which are then processed into bilirubin, and Fe ions and bilirubin inhibit NF-κB expression. HO-1 = heme oxygenase 1, Keap1 = Kelch-like ECH-associated protein, NQO1 = NADPH quinone oxidoreductase 1, Nrf2 = nuclear factor erythroid 2-related factor 2

In the quiescent state, Keap1 binds to Nrf2, curbing its activity through ubiquitination and proteasomal degradation.^[92]^ The onslaught of oxidative stress activates the Nrf2/HO-1 pathway; Keap1 undergoes dimerization and rendezvous with CUL-3, facilitating Nrf2’s combination with small Maf proteins.^[93,94]^ This combine forges a transcriptionally active heterodimer that ventures into the nucleus, allies with ARE, and spearheads the transcription of antioxidant bastions, including NQO1 and HO-1, thus staging a formidable stand against oxidative stress.^[94,95]^ HO-1 catalyzes the breakdown of heme into free iron ions, carbon monoxide, and biliverdin, subsequently transformed into bilirubin. Both Fe^2+^ and bilirubin orchestrate the suppression of NF-κB expression,^[96]^ as illustrated in Figure 4. Additionally, Nrf2 can neutralize NF-κB-mediated inflammatory responses by competitively binding to CBP^[97,98]^ and by reducing NLRP3 expression through caspase-1 cleavage, thereby lowering IL-1β levels and inhibiting neuroinflammation.^[99]^ Nrf2 expression, therefore, emerges as a strategic bulwark to dampen NF-κB and NLRP3 expression via the Nrf2/HO-1 pathway, heralding a reduction in neuroinflammation.

### 2.3. Peripheral inflammation: a catalyst for CNS involvement in PD

**Figure 5.**
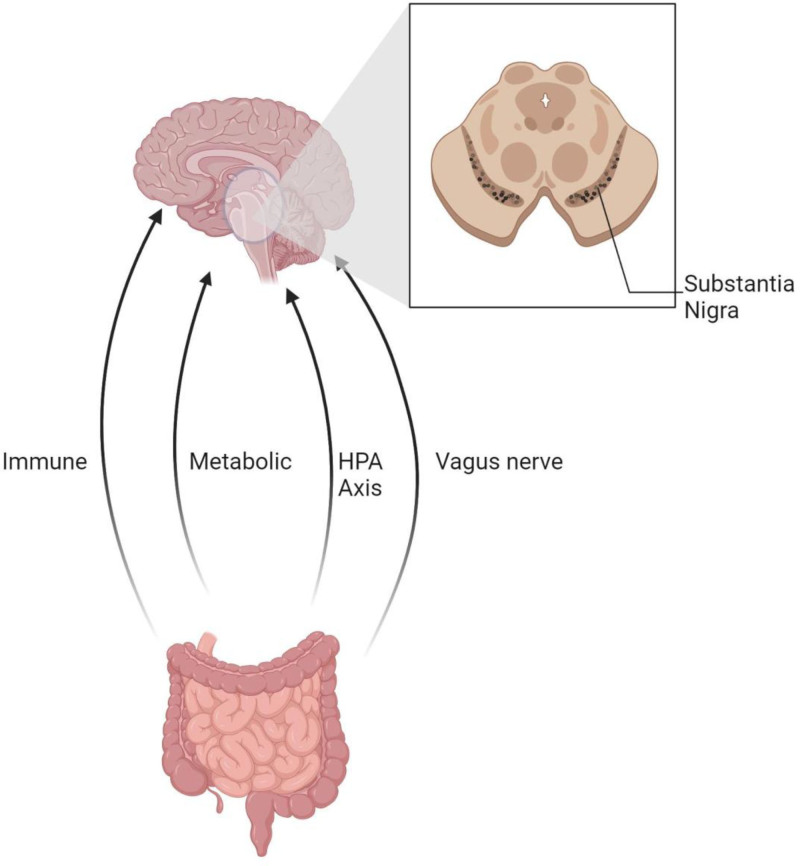
The GBA in PD. The GBA in PD including the VN, immune mechanisms, metabolic processes, and the endocrine system. GBA = gut-brain axis, PD = Parkinson disease, VN = vagus nerve.

Peripheral inflammation, particularly within the intestinal tract, stands as a critical trigger for cascading effects within the CNS in PD. This phenomenon, mediated through the intricate GBA, underscores the profound influence of intestinal health on neuroinflammation.^[100]^ Central to this axis is the gut microbiome, which, in synergy with intestinal epithelial cells, safeguards the integrity of the intestinal barrier.^[101]^ This dynamic communication network between the brain and the gut microbiota is facilitated by a myriad of pathways including the VN, immune mechanisms, metabolic processes, and the endocrine system, highlighting the complexity and bidirectionality of these interactions,^[102]^ as illustrated in Figure 5.

#### 2.3.1. The GBA in PD

①Vagal and Spinal Pathways: Groundbreaking autopsies by Braak et al have illuminated the pivotal role of the VN, emanating from the dorsal motor nucleus of the medulla, in the gut-brain communication network.^[103]^ This vital conduit offers a dual regulatory mechanism over the gastrointestinal tract, gut signals enabling direct transmission to the brain via the enteric nervous system and neuroendocrine links, intricately woven into the fabric of the gut-brain axis.^[104,105]^

②Immunological Cross-Talk: The activation of immune cells within the gut modulates neuronal activity through the release of neurotransmitters and cytokines, which are then ferried to the CNS via the VN. This immunological brigade, comprising T cells, B cells, dendritic cells, and macrophages,^[106]^ launches pro-inflammatory cytokines like TNF-α, IFN-γ, IL-1β, and IL-6^[107]^ into the CNS through the gut-brain conduit, igniting neuroinflammation.^[108]^

③Metabolic Interplay: The metabolic contribution, highlighted by SCFAs and bile acids, emerges from the fermentation of dietary fibers by gut microbiota. These metabolic byproducts activate enteroendocrine cells, establishing a communication channel with the CNS.^[109]^

④Endocrine Dynamics: The HPA axis serves as a critical endocrine pathway facilitating this complex dialogue.^[110]^

#### 2.3.2. Inflammation’s journey through the GBA

**Figure 6.**
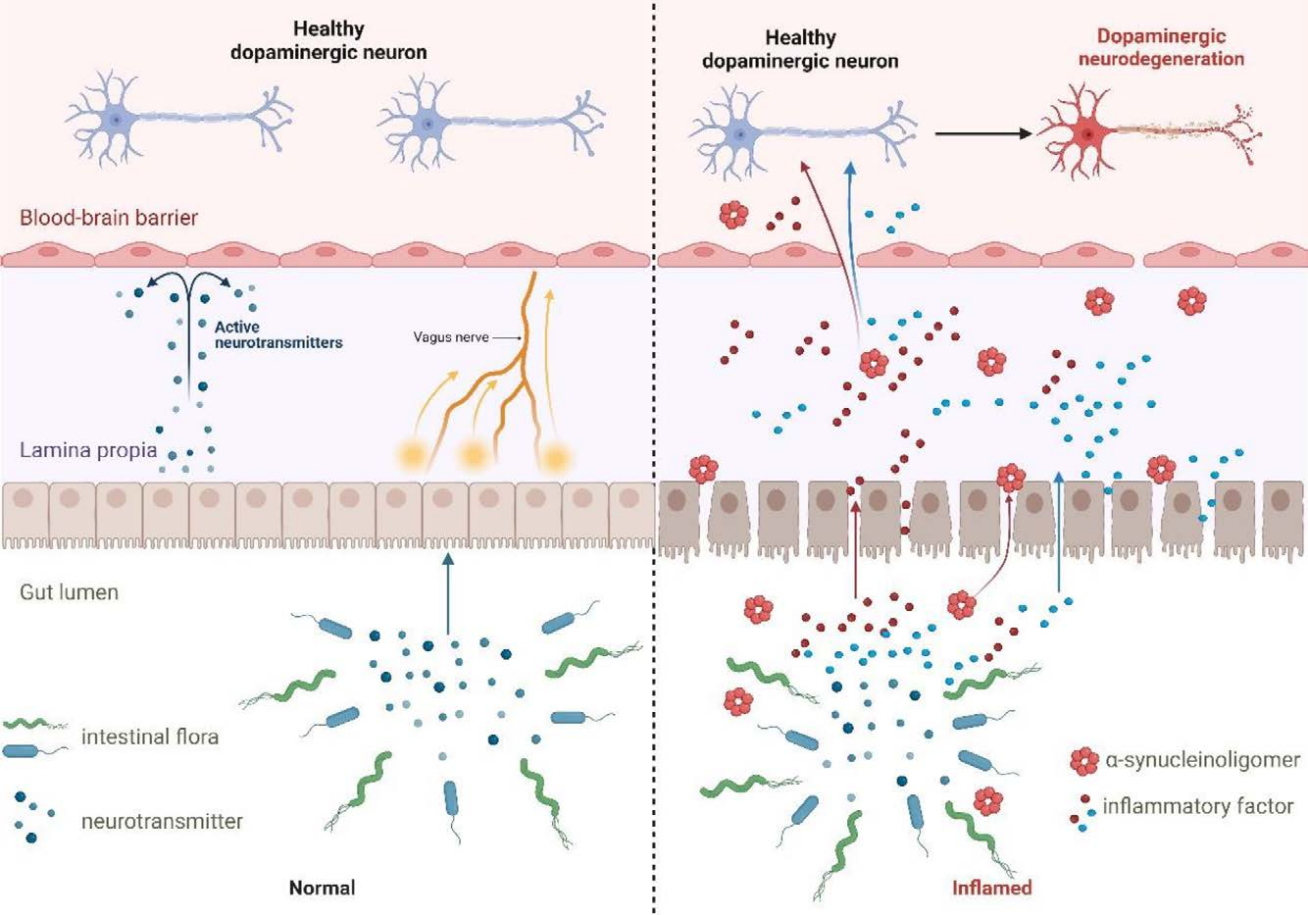
Inflammation in the GBA. Physiologically, the intestinal barrier is intact with the blood-brain barrier, and various factors are unable to enter the CNS through the gut-brain axis. Toxic substances such as MPTP, 6-OHDA, LPS, rotenone, and oligomerized α-synuclein cause dysbiosis of the gut microbiota, disruption of the integrity of the intestinal barrier, and an increase in intestinal permeability, which promotes both intestinal inflammation and systemic peripheral inflammation. Intestinal inflammation through the gut-brain axis stimulates the central nervous system, destroying the blood-brain barrier, and pro-inflammatory cytokines IL-1β, TNF-α, IFN-γ, IL-6, etc, and activated immune cells in the peripheral circulation to enter the brain, causing neuroinflammation. CNS = central nervous system, GBA = gut-brain axis, IFN-γ = interferon-γ.

The integrity of the intestinal and BBB underpins the selective permeability to the CNS via the GBA. These barriers, orchestrated by intestinal epithelial cells and regulated by tight junction proteins like occludin and ZO-1, are the gatekeepers against unwarranted intrusions.^[111,112]^ However, disruptive agents such as MPTP, 6-OHDA, LPS, and Rotenone, alongside aggregates of α-synuclein,^[113]^ can unsettle the gut microbiota balance, compromise the intestinal barrier, and heighten permeability, thus fostering intestinal and systemic inflammation.^[103]^ This process not only breaches the CNS’s protective barriers but also ushers pro-inflammatory agents and immune cells into the brain, precipitating neuroinflammation,^[107,114]^ as illustrated in Figure 6. Furthermore, persistent intestinal inflammation may fuel the accumulation of α-synuclein, which can navigate through the GBA to the SNpc, potentially triggering PD.^[115–117]^ Therefore, addressing peripheral intestinal inflammation stands as a promising avenue to mitigate neuroinflammation, offering a pathway to therapeutic interventions in PD.

### 2.4. Autoimmunity

Autoimmunity, arising from the dysregulation of innate and adaptive immune responses, is linked to inflammation in PD.^[118]^ Autoimmune diseases (AIDs) are characterized as conditions in which the host mounts an immune response against its own tissues, leading to pathological states due to the resultant tissue damage. Patients with AIDs exhibit autoantibodies or autoreactive, antigen-specific T cells that propel the progression of the disease.^[119]^ Central to the autoimmune response in PD is SNCA.^[120]^ The SNCA gene is responsible for encoding alpha-synuclein, mutations of which lead to its aggregation. These aggregates activate microglia, triggering them to release inflammatory cytokines such as IL-1β and IL-6 via the NF-κB pathway.^[121]^ Additionally, microglia play a crucial role in antigen presentation, processing and presenting endogenous or ingested protein fragments through MHC-II molecules, essential for triggering specific immune responses and facilitating the activation of CD4 + T cells. These activated CD4 + T cells release various pro-inflammatory cytokines, including IFN-γ and TNF-α, intensifying the inflammatory response and potentially prompting further migration of immune cells to the central nervous system, thereby exacerbating the disease. Moreover, microglia may also activate CD8 + T cells via the MHC-I pathway. As cytotoxic T cells, CD8 + T cells can directly destroy neurons that are compromised by protein aggregation or other abnormalities. These immune reactions amplify neuronal damage and neurodegenerative alterations. The activation of CD4 + T cells further attracts B cells and other T cell subsets, amplifying the immune response and aggravating the inflammatory milieu in PD.^[122]^ The management of PD-related inflammation driven by autoimmunity is still under investigation. Nonetheless, based on the described mechanisms, strategies targeting microglia, employing immune therapies that involve CD4 + and CD8 + T cells, reducing levels of TNF-α and IFN-γ, and suppressing MHC-II-mediated antigen presentation represent potential therapeutic approaches for addressing autoimmunity-induced inflammation in PD.^[121]^

## 3. Active ingredients in TCM for PD inflammation

Chinese medicine has been utilized for a long history in treating PD, from which we can draw some experience for our better treatment of PD. However, due to the numerous components and unknown specific pathways of Chinese medicine’s onset of action, we still need to study them, and here we summarize 42 active ingredients in Chinese medicines for the treatment of inflammation in PD and introduce the potential mechanisms by which they may work.

### 3.1. Polyphenol compounds

As secondary metabolites, polyphenols are found in plant kingdom (herbs, plants, fruits, vegetables, grains, tea, coffee, and other plants).^[123]^ Natural flavonoids are a type of polyphenol. Polyphenols are effective in relieving neuroinflammation, as illustrated in Table 1.

**Table 1:** Polyphenol compounds for PD neuroinflammation.

Name	Experimental PD models	Effects on inflammation	Effects on cytokine levels	Reference
Hyperoside	BV2 cells from LPS-induced mice	Microglial activation, p38/MAPK, NF-κB↓	Iba-1, TNF-α, IL-6, NO, iNOS↓	^[124]^
Quercetin	LPS-induced mice	Microglia activation, NF-κB/NLRP3↓	Iba-1, NLRP3, Caspase-1, IL-1β↓	^[125]^
Curcumin	Rotenone-induced mice	Microglia activation, NF-κB/ NLPR3↓	Iba-1, NF-κB, NLPR3, caspase-1, IL-1β↓	^[126]^
Resveratrol	6-OHDA-induced rats	Neuroinflammation↓	TNF-α、COX-2↓	^[127]^
	Rotenone-induced rats	Microglial activation↓	IL-1β↓	^[128]^
Icariin	6-OHDA-induced rats	Microglial activation, NF-κB/NLRP3↓	Iba-1, NO, iNOS, TNF-α, IL-1β↓	^[129]^
Apigenin	Rotenone-induced rats	NF-κB↓	TNF-α, IL-6, iNOS↓	^[130]^
Kaempferol	6-OHDA-induced rats	Microglial activation, Astrocytic activation, p38/MAPK/NF-κB, NF-κB/NLRP3↓	Iba-1, GFAP, IL-1β, IL-18, COX-2, iNOS↓	^[131,132]^
	Rotenone-induced rats	Neuroinflammation↓	IL-6, TNF-α↓	^[133]^
Catechins	Rotenone-induced rats	NF-κB↓	IκKB, IL-1β, IL-6, TNF-α, NO↓	^[134,135]^
Luteolin	LPS-induced rats	Microglial activation↓	TNF-α, NO↓	^[136]^
Farrerol	LPS-induced rats	Microglial activation, MAPK/Akt/NF-κB↓	Iba-1, iNOS, TNF-α, IL-1β, IL-6↓	^[137,138]^
Galangin	LPS-induced rats	Microglial activation, MAPK/Akt/NF-κB↓	Iba-1, IL-1β, IL-6, TNF-α, COX-2, iNOS↓	^[139]^
Hesperidin	6-OHDA-induced mice	Neuroinflammation↓	IL-1β, IL-2, IL-6, IFN-γ, TNF-α↓	^[140,141]^
Isoliquiritigenin	BV2 cells from MPTP-induced mice	Microglial activation, JNK/NFκB/Akt↓	Iba-1, iNOS, COX-2↓	^[142]^
	BV2 cells from LPS-induced mice	JNK/NFκB/Akt↓	TNF-α, IL-1β, IL-6↓	^[142]^
Rutin	6-OHDA-induced rats	Neuroinflammation↓	IL-1, TNF-α, NO, iNOS↓	^[143]^
Ferulic Acid	Rotenone-induced rats	Microglial activation, Astrocytic activation↓	Iba-1, GFAP, IL-1β, IL-6, TNF-α, COX-2, iNOS↓	^[144,145]^
Phloretin	MPTP-induced mice	Microglial activation, Astrocytic activation, NF-κB↓	Iba-1, GFAP, iNOS, COX-2, IL-1β, TNF-α↓	^[146,147]^
Morin	MPTP-induced mice	Microglial activation, Astrocytic activation, ERK/MAPK↓	Iba-1, GFAP, NF-κB↓	^[148]^
Mangiferin	6-OHDA-induced rats	NF-κB, Th1↓, Th2↑	IL-1β, IL-6, TNF-α, COX-2, IFN-γ↓, IL-4↑	^[149,150]^
Nobiletin	MPTP-induced rats	Microglial activation↓	Iba-1, IL-1β↓	^[151,152]^

PD = Parkinson disease.

Hyperoside is a flavonoid active ingredient extracted from the Chinese herbs Acanthopanax senticosus and Hypericum perforatum. Hypericum perforatum is able to reduce the release of TNF-α, IL-6, NO and iNOS in LPS-stimulated BV2 cells by inhibiting the p38/MAPK and NF-κB pathways and reducing the nuclear translocation of p65, thereby downregulating the expression of microglia and attenuating neuroinflammation.^[124]^ Quercetin, a flavonoid compound, is extensively found in fruits, vegetables, and other plants. In PD mouse models induced by LPS, quercetin alleviates neuroinflammation and safeguards dopaminergic neurons by reducing Iba-1 expression in the SNpc, curtailing microglial activation, and repressing the NF-κB/NLRP3 pathway. This action inhibits NLRP3 and Caspase-1 activity and lowers IL-1β levels.^[125]^

#### 3.1.1. Curcumin

Curcumin, a natural polyphenolic compound, is derived from the rhizomes of plants within the ginger family, such as turmeric and zedoary. In PD mice induced by rotenone, curcumin suppresses Iba-1 expression, inhibits microglial activation, dampens the NF-κB/NLPR3 pathway, diminishes NF-κB activity, and restrains the activation of NLPR3 and caspase-1. Consequently, it reduces the discharge of IL-1β.^[126]^

#### 3.1.2. Resveratrol

Resveratrol, a polyphenolic organic substance, is extractable from *Polygonum cuspidatum* and grapes. In 6-OHDA-induced rat models of PD, resveratrol lowers the concentrations of TNF-α and COX-2 in the SNpc, mitigates neuroinflammation, protects dopaminergic neurons, and ameliorates the aberrant rotational behavior in mice.^[127]^ In rotenone-induced PD rats, resveratrol blocks microglial activation, lessens IL-1β levels, and suppresses neuroinflammation.^[128]^

#### 3.1.3. Icariin

Icariin is a flavonoid compound that can be found in the stems and leaves of Epimedium. In PD rats, icariin has been shown to downregulate the levels of Iba-1, inhibit the activation of microglia, and decrease the release of IL-1β, TNF-α, NO, and iNOS by inhibiting the expression of the NF-κB/NLRP3 pathway. This action alleviates neuroinflammation and protects dopaminergic neurons.^[129]^

#### 3.1.4. Apigenin

Apigenin is a flavonoid compound found in temperate and tropical regions, particularly abundant in celery. In PD rats induced by rotenone, apigenin reduces neuroinflammation by inhibiting the levels of NF-κB, thereby decreasing the levels of TNF-α, IL-6, NF-κB, and iNOS-1 in the striatum.^[130]^

#### 3.1.5. Kaempferol

The flavonoid compound kaempferol is widely found in tea leaves, broccoli, snapdragons, witch hazel, grapefruits, brussels sprouts, and apples. In PD rat models induced by 6-OHDA, kaempferol can inhibit the expression of GFAP and Iba-1, suppress the microglial activation and the astrocytic activation via the NF-κB/NLRP3 and p38/MAPK/NF-κB pathways, and decrease the concentrations of iNOS, COX-2, IL-1β, and IL-18, thereby inhibiting neuroinflammation.^[131,132]^ In PD rats induced by rotenone, kaempferol also decreases the level of TNF-α and IL-6, protecting dopaminergic neurons and alleviating the motor deficits of PD rats.^[133]^

#### 3.1.6. Catechins

Catechins, a class of phenolic compounds, are extracted from natural plants such as tea leaves and fruits. In PD rats induced by rotenone, catechins can reduce the content of IκKB, thereby decreasing the concentrations of IL-1β, IL-6, TNF-α, and NO by downregulating NF-κB pathway, thus inhibiting neuroinflammation.^[134,135]^

#### 3.1.7. Luteolin

Luteolin, a flavone subclass of flavonoids belonging to the natural polyphenolic compound, is found in plant-based foods. In PD rats induced by LPS, luteolin can inhibit microglial activation, reduce the release of TNF-α and NO, suppress neuroinflammation, and protect dopaminergic neurons.^[136]^

#### 3.1.8. Farrerol

Farrerol is a flavonoid compound extracted from the Rhododendron genus, specifically *Rhododendron simsii*.^[137]^ In rats afflicted with PD induced by LPS, farrerol mitigates the microglial activation by suppressing the level of Iba-1 via downregulating the expression of the MAPK/AKT/NF-κB pathway, decreasing the expression of TNF-α, IL-1β, IL-6, and iNOS, thus preventing the loss of dopaminergic neurons and easing motor dysfunction in PD rats.^[138]^

#### 3.1.9. Galangin

Galangin, a flavonoid compound, is extensively found in the roots of the ginger family plant Alpinia galanga. In PD rats, galangin significantly reduces Iba-1 expression and suppress the activation of microglia. Downregulating the expression of the MAPK/Akt/NF-κB pathway decreases the release of COX-2, iNOS, TNF-α, IL-1β, and IL-6, thereby suppressing neuroinflammation.^[139]^

#### 3.1.10. Hesperidin

Hesperidin, a bioflavonoid with a high concentration in citrus fruits,^[140]^ can reduce the release of TNF-α, IFN-γ, IL-1β, IL-2, and IL-6 in the striatum of PD mouse models induced by 6-OHDA, inhibiting neuroinflammation, protecting dopaminergic neurons, and alleviating depressive, anxious, and dyskinetic behaviors in PD mice.^[141]^

#### 3.1.11. Isoliquiritigenin

Isoliquiritigenin, extracted as a chalcone compound from licorice, targets BV2 cells from MPTP-induced PD mice. It reduces the expression of Iba-1, curbing the microglial activation. It lowers the concentration of COX-2 and iNOS by suppressing the JNK/NFκB/AKT pathway. In addition, in PD mice triggered by LPS, isoliquiritigenin decreases the expression of TNF-α, IL-1β, and IL-6 via the JNK/NFκB/AKT pathway, reducing neuroinflammation and safeguarding dopaminergic neurons.^[142]^

#### 3.1.12. Rutin

Rutin, a quercetin derivative and part of the flavonoid family, demonstrates neuroprotective properties in PD rat models induced by 6-OHDA. It lessens the level of NO, iNOS, TNF-α, and IL-1β, thereby reducing neuroinflammation and preserving dopaminergic neurons. As a result, rutin diminishes rotational behavior and motor deficits in PD rats.^[143]^

#### 3.1.13. Ferulic acid

Ferulic Acid is a phenolic substance derived from the traditional Chinese herb Ferula.^[144]^ In PD models induced by rotenone in rats, Ferulic Acid effectively curtails the levels of GFAP and Iba-1, mitigates the activation of microglia and astrocytes, respectively. This action leads to a notable downregulation in the striatal concentrations of TNF-α, IL-1β, IL-6, COX-2, and iNOS, thereby mitigating neuroinflammation and safeguarding dopaminergic neurons.^[145]^

#### 3.1.14. Phloretin

Phloretin, a dihydrochalcone compound prevalent in the peels and root bark of fruits like apples and pears,^[146]^ plays a pivotal role in neuroprotection within PD mouse models triggered by MPTP. By downregulating Iba-1 and GFAP, Phloretin inhibits the microglial and astrocytic activation. The suppression of the NF-κB pathway underlies its ability to diminish the release of iNOS, COX-2, TNF-α, and IL-1β, contributing to the protection of dopaminergic neurons. Additionally, Phloretin boosts balance and prolongs retention time while alleviating orientation impairments in PD mice.^[147]^

#### 3.1.15. Morin

Morin is extracted from the bark of Morus alba and other members of the Moraceae family, presenting flavonoid-based therapeutic potential against PD. In MPTP-induced PD mouse models, Morin downregulates GFAP and Iba-1 expressions, thus impeding the microglial and astrocytic activation. By suppressing the ERK/MAPK pathway and reducing NF-κB expression, Morin effectively combats neuroinflammation.^[148]^

#### 3.1.16. Mangiferin

Mangiferin, a compound rich in xanthonoids and primarily harvested from mango trees, stands out for its potency.^[149]^ In PD rat models induced by 6-OHDA, Mangiferin showcases its therapeutic might by suppressing NF-κB expression. This action significantly diminishes the expressions of COX-2, IL-1β, IL-6, and TNF-α. It also targets and reduces the levels of Th1 and Th2 cells, further lowering the amounts of IFN-γ and increasing the amounts of IL-4, thereby robustly countering neuroinflammation.^[150]^

#### 3.1.17. Nobiletin

Nobiletin, a naturally occurring flavonoid in citrus fruits, plays a critical role in neuroprotection within PD models induced by MPTP in rats. By targeting and downregulating Iba-1 expression in the substantia nigra, it prevents microglial activation and decreases IL-1β levels, effectively preserving the integrity of dopaminergic neurons.^[151,152]^

### 3.2. Terpenoids

Terpenoids are the most diverse natural products, categorized as monoterpenes, diterpenes, triterpenes, tetraterpenes, polyphenols, etc, and have anti-inflammatory activity,^[153]^ as illustrated in Table 2.

**Table 2:** Terpenoids for PD neuroinflammation.

Name	Experimental PD models	Effects on inflammation	Effects on cytokine levels	Reference
Andrographolide	MPTP-induced mice	Microglia activation, NF-κB/NLRP3↓	Iba-1, IκBα, NLRP3, Caspase-1, IL-1β, TNF-α↓	^[154–156]^
Astragaloside IV	MPTP-induced mice	Microglia activation, NF-κB/NLRP3↓	Iba-1, NF-κB, p65, NLRP3, IL-1β↓	^[157]^
	SH-SY5Y cells from 6-OHDA-induced mice	JAK2/STAT3↓	TNF-α, IL-6, iNOS↓	^[158]^
Paeoniflorin	MPTP-induced mice	Microglial activation, Astrocytic activation↓	CD11b, GFAP, IL-1β, TNF-α, NO, iNOS↓	^[159,160]^
Tanshinone I	MPTP-induced mice	Microglia activation, NF-κB↓	Iba-1, TNF-α↓, IL-10↑	^[161,162]^
	BV2 cells from LPS-induced mice	Neuroinflammation↓	TNF-α, IL-6, IL-1β, iNOS↓	^[162]^
Tanshinone IIA	6-OHDA-induced rats	NF-κB↓	IL-1β, TNF-α, INF-γ↓, IL-10↑	^[163]^
	MPTP-induced mice	Microglial activation↓	CD11b, iNOS↓	^[164]^
Ginsenoside Rg1	MPTP-induced mice	Microglial activation, CD3, CD8 T cells in peripheral blood, CD3 T cells in SNpc↓, CD3, CD4 T cells in peripheral blood↑	Iba-1, TNF-α, IFN-γ, IL-1β, IL-6, α-synuclein↓	^[165–167]^
	LPS-induced mice	Microglial activation, Astrocytic activation, NF-κB↓	Iba-1, GFAP, TNF-α, IL-1β, IL-6↓, TGF-β, IL-10↑	^[168]^
Ginsenoside Rb1	LPS-induced mice	Microglial activation, Astrocytic activation, NF-κB↓	Iba-1, TNF-α, IL-1β, iNOS, COX-2↓, IL-10, TGF-β↑	^[169,170]^
Menthol	LPS-induced rats	Microglial activation, MAPK/AKT/NF-κB↓	Iba-1, COX-2, iNOS, TNF-α, IL-6, IL-1β↓	^[171]^
Ginkgolide K	MPTP-induced mice	Microglial activation, Astrocytic activation↓	Iba-1, GFAP, NO, IL-1β, TNF-α↓, IL-10, TGF-β↑, peripheral IFN-γ, IL-17↓	^[172]^
Ginkgolide B	MPTP-induced mice	Microglial activation, Astrocytic activation, TLR4↓	TNF-α, iNOS↓	^[173]^
Triptolide	LPS-induced rats	Microglial activation, Astrocytic activation↓	Iba-1, GFAP, iNOS, TNF-α、IL-1β, IL-6↓	^[174,175]^
Glycyrrhizin	MPTP-induced mice	Microglial activation, Astrocytic activation↓ Combined with High Mobility Group Box Protein 1 (HMGB1)	Iba-1, GFAP, TNF-α↓	^[176,177]^
	Rotenone-induced rats	Microglial activation, Astrocytic activation↓	GFAP, Iba-1, IL-1β, IL-6, TNF-α, COX-2, iNOS↓	^[178]^
	MPTP-induced Danio rerio	TLR/NFκB↓, Combined with HMGB1	IL-1β, IL-6, α-synuclein↓	^[179]^
Cannabinoids	LPS-induced mice	Microglial activation↓	Iba-1, TNF-α, IL-1β, iNOS↓	^[180]^
	MPTP-induced mice	Microglial activation↓	Iba-1, TNF-α, IL-1β↓	^[181]^

PD = Parkinson disease.

#### 3.2.1. Andrographolide

Andrographolide, a terpenoid derived from the *Andrographis paniculata* herb, showcases profound neuroprotective properties in PD mouse models induced by MPTP. This compound achieves its effects through downregulating the NF-κB/NLRP3 pathway, leading to reduced Iba-1 expression, minimized microglial activation, and lowered NLRP3 and Caspase-1 activity. Consequently, there’s a notable decrease in IL-1β levels, effectively dampening neuroinflammation, safeguarding dopaminergic neurons, and alleviating motor deficits seen in PD mice.^[154]^ Moreover, andrographolide impedes the activity of p65 and suppresses IκBα expression, thus inhibiting the levels of the NF-κB pathway triggered by TNF-α and IL-1β, further reducing neuroinflammation and enhancing both neuronal protection and motor function recovery in PD mice.^[155,156]^

#### 3.2.2. Astragaloside IV

Astragaloside IV, identified as a tetracyclic triterpenoid saponin from Astragali Radix, demonstrates potential therapeutic effects in MPTP-induced PD mouse models. It effectively downregulates Iba-1 expression, halts microglial activation, and prevents NLRP3 inflammasome activation. By curtailing phosphorylated nuclear NFκB/p65 levels and obstructing the NF-κB/NLRP3 pathway, Astragaloside IV reduces NLRP3 and IL-1β concentrations, thus protecting dopaminergic neurons and reducing motor impairments.^[157]^ Additionally, in SH-SY5Y cells from the PD model induced by 6-OHDA, Astragaloside IV inhibits the JAK2/STAT3 pathway, decreasing TNF-α, IL-6, and iNOS levels, which helps in mitigating neuroinflammation.^[158]^

#### 3.2.3. Paeoniflorin

Paeoniflorin, extracted from *Paeonia lactiflora* and *Paeonia veitchii*, is a monoterpenoid glycoside with notable neuroprotective capabilities in MPTP-induced PD mouse models. It downregulates CD11b and GFAP expression, inhibiting microglial and astrocytic activation, leading to reduced IL-1β, TNF-α, NO, and iNOS levels. This multifaceted approach helps protect dopaminergic neurons and enhances motor function in PD mice.^[159,160]^

#### 3.2.4. Tanshinone

Tanshinone is a diterpenoid derived from *Salvia miltiorrhiza*, and the main active ingredients are tanshinone I and tanshinone IIA.^[161]^ Tanshinone I showcases its neuroprotective prowess in MPTP-induced PD mouse models by reducing Iba-1 expression and inhibiting microglial activation. It also downregulates the NF-κB pathway transcription, decreases TNF-α levels, and increases IL-10 levels. Furthermore, in LPS-induced PD mouse BV2 cells, tanshinone I lowers TNF-α, IL-6, IL-1β, and iNOS levels, effectively countering neuroinflammation.^[162]^ In 6-OHDA-induced PD rat, tanshinone IIA mitigates NF-κB activity, reduces IL-1β, TNF-α, and INF-γ levels, and increases IL-10 levels, thereby alleviating neuroinflammation and easing abnormal rotation and muscle rigidity.^[163]^ Tanshinone IIA also diminishes CD11b expression in the SNpc, curtails microglial activation, and decreases iNOS levels in MPTP-induced PD mice, further reducing neuroinflammation.^[164]^

#### 3.2.5. Ginsenosides

Ginsenosides, identified as triterpene glycosides and extracted from ginseng, with over 500 variants recognized, play a significant role in PD treatment.^[165]^ Specifically, ginsenoside Rg1 in MPTP PD models boosts CD3 and CD4 T cell levels in peripheral blood, lessens CD3 and CD8 T cell levels, and reduces the infiltration of CD3 T cells in the SNpc region. It inhibits T cell cytotoxicity and downregulates Iba-1 expression, thereby suppressing microglial activation, lowering the expression of IL-1β, IL-6, TNF-α, and IFN-γ, and preventing α-synuclein aggregation.^[166,167]^ In LPS-induced PD mice, ginsenoside Rg1 was able to downregulate GFAP and Iba-1, inhibit the microglial activation and the astrocytic activation, reduce the concentrations of TNF-α, IL-1β, and IL-6, by downregulating the levels of the NF-κB pathway, increase the concentrations of TGF-β and IL-10, and reduce neuroinflammation.^[168]^ Ginsenoside Rb1 in LPS PD mice by downregulates the levels of GFAP and Iba-1, inhibits microglial and astrocyte activation via the NF-κB pathway, increases IL-10 and TGF-β levels, and reduces IL-1β and TNF-α levels, mitigating neuroinflammation.^[169,170]^

#### 3.2.6. Menthol

Menthol, a cyclic monoterpene found in mint leaves and stems, in LPS-induced PD rats, attenuates IBA-1 expression, inhibits the activation of microglia, and via the MAPK/AKT/NF-κB pathway, reduces IL-1β, IL-6, TNF-α, iNOS, and COX-2 levels. These actions protect dopaminergic neurons and lessen abnormal movement in PD rats.^[171]^

#### 3.2.7. Ginkgolides

Ginkgolides, distinguished terpenoid compounds within ginkgo leaves, have captured significant scientific interest, particularly Ginkgolide B and K. Ginkgolide K, in MPTP PD models, orchestrates a reduction in GFAP and Iba-1 expression, curtails the microglial activation and the astrocytic activation, thereby diminishes NO, TNF-α, and IL-1β levels while enhancing TGF-β and IL-10 concentrations. Additionally, it attenuates peripheral inflammatory cytokines like IFN-γ and IL-17, effectively mitigating neuroinflammation, curbing the depletion of dopaminergic neurons, and fostering the transformation of astrocytes into dopaminergic neurons.^[172]^ Ginkgolide B further demonstrates its neuroprotective efficacy by inhibiting TLR4 expression on microglia and astrocytes, reducing TNF-α levels, and suppressing iNOS expression in activated microglia.^[173]^

#### 3.2.8. Triptolide

Triptolide, a potent compound from *Tripterygium wilfordii*, also known as Thunder God Vine, has shown neuroprotective effects in LPS-induced rat models of PD. By diminishing the levels of markers Iba-1 and GFAP, triptolide effectively curtails the microglial activation and the astrocytic activation, lowers the levels of neuroinflammatory mediators iNOS, TNF-α, IL-1β, and IL-6. Through these mechanisms, triptolide suppresses neuroinflammation, safeguarding dopaminergic neurons and offering a potential therapeutic avenue for PD.^[174,175]^

#### 3.2.9. Glycyrrhizin

Glycyrrhizin, a triterpenoid compound extracted from licorice, a staple in TCM, demonstrates significant anti-inflammatory properties. The activation of microglial TLR2/TLR4 receptors by HMGB1 initiates a series of responses that include both MyD88-dependent and independent pathways, along with MAPKs and NLRP3, among others. This cascade results in the transcription of inflammatory genes and the subsequent microglial activation.^[176]^ In MPTP-induced PD mice, glycyrrhizin’s binding to HMGB1 leads to a reduction in GFAP and Iba-1 levels, curtails the microglial activation and the astrocytic activation, lowers TNF-α levels, and effectively dampens neuroinflammation.^[177]^ Likewise, in a PD rat induced by rotenone, glycyrrhizin mitigates neuroinflammation by suppressing the levels of GFAP and Iba-1, reducing the activation of microglia and astrocytes, and decreasing the concentrations of iNOS, COX-2, TNF-α, IL-1β, and IL-6. This series of actions helps safeguard dopaminergic neurons.^[178]^ Moreover, in MPTP PD zebrafish, glycyrrhizin’s interaction with HMGB1 and modulation of the TLR/NFκB pathway downregulates the release of IL-1β and IL-6, prevention of abnormal α-synuclein aggregation, inhibition of neuroinflammation, protection of dopaminergic neurons, and amelioration of motor impairments.^[179]^

#### 3.2.10. Cannabinoids

Cannabinoids, belonging to the terpenophenolic compound family, have shown promising results in LPS-induced PD mice by diminishing Iba-1 expression and inhibiting microglial activation. This leads to a downregulation in the release of iNOS, IL-1β, and TNF-α thereby alleviating neuroinflammation.^[180]^ In the context of PD mouse models induced by MPTP, cannabinoids continue their protective role by lowering Iba-1 expression, inhibiting the activation of microglia, and consequently reducing TNF-α and IL-1β release. This concerted action preserves dopaminergic neurons and ameliorates motor impairments observed in PD mice.^[181]^

### 3.3. Other types of compounds

As illustrated in Table 3.

**Table 3:** Other types of compounds for PD neuroinflammation.

Name	Experimental PD models	Effects on inflammation	Effects on cytokine levels	Reference
Berberine	LPS-induced mice	NF-κB↓	IL-6, TNF-α↓	^[182]^
Piperine	6-OHDA-induced rats	Neuroinflammation↓	TNF-α, IL-1β↓	^[183]^
	Rotenone-induced rats	Neuroinflammation↓	TNF-α, IL-1β, IL-6↓	^[184]^
Salidroside	MPTP-induced mice	TLR4/NF-κB, NLRP3/Caspase-1↓	NLRP3, Caspase-1, IL-1β, IL-18, α-synuclein↓	^[185,186]^
Octanol	LPS-induced rats	Microglial activation↓	CD11b, TNF-α, iNOS, NO↓	^[187]^
α-Asarone	MPTP-induced mice	Microglial activation, Astrocytic activation, NF-κB↓	Iba-1, GFAP, iNOS, COX-2↓	^[188]^
Osthole	MPTP-induced mice	Microglial activation↓	Iba-1, TNF-α, IL-6, IL1β, NO↓	^[189]^

PD = Parkinson disease.

#### 3.3.1. Berberine

Berberine, hailing from the traditional Chinese herb Coptis chinensis, is an isoquinoline alkaloid with pronounced neuroprotective effects in MPTP PD models. It adeptly navigates the NF-κB pathway to diminish IL-6 and TNF-α levels, effectively easing neuroinflammation and providing a shield for neurons.^[182]^

#### 3.3.2. Piperine

Piperine, a potent alkaloid present in the fruits of the Piperaceae family, notably black pepper, showcases therapeutic promise in 6-OHDA PD models. It lowers TNF-α and IL-1β levels, curtails neuroinflammation, and fortifies dopaminergic neurons, enhancing the rotational behavior of PD rats.^[183]^ Further, in rotenone-induced PD rats, piperine, in concert with quercetin, attenuates TNF-α, IL-1β, and IL-6 levels, thwarting the degeneration of dopaminergic neurons, refining narrow beam walking parameters, and boosting motor vitality.^[184]^

#### 3.3.3. Salidroside

Salidroside, derived from the Rhodiola genus roots, emerges as a promising agent against PD.^[185]^ In MPTP-induced PD mice, salidroside exhibits mechanism involves downregulating the TLR4/NF-κB and NLRP3/Caspase-1 pathways, leading to reduced activity of NLRP3 and Caspase-1. Consequently, the production of IL-1β and IL-18 by NLRP3 inflammasomes is diminished, alongside a notable decrease in α-synuclein aggregation. These actions collectively contribute to the suppression of neuroinflammation and enhancement in motor impairments observed in PD mice.^[186]^

#### 3.3.4. Octanol

Derived from the Chinese herb *Rehmannia glutinosa*, Octanol is a saturated fatty alcohol that exhibits neuroprotective capabilities in LPS-induced PD rat models. By reducing CD11b expression and staving off microglial activation, it decreases TNF-α, iNOS, and NO levels, thereby suppressing neuroinflammation.^[187]^

#### 3.3.5. α-Asarone

α-Asarone, extracted from the *Acorus calamus*, is a phenylpropene compound with notable neuroprotective attributes in MPTP PD models. It impedes the concentrations of Iba-1 and GFAP, inhibits microglial and astrocyte activation via the NF-κB pathway, and lessens COX-2 and iNOS levels, thus safeguarding dopaminergic neurons and curtailing neuroinflammation.^[188]^

#### 3.3.6. Osthole

Osthole, a coumarin derivative sourced from *Cnidium monnieri*, serves as a neuroprotective agent in MPTP-induced PD mice. It inhibits Iba-1 expression, dampens microglial activation, and reduces TNF-α, IL-6, IL-1β, and NO levels, effectively mitigating neuroinflammation.^[189]^

### 3.4. Active ingredients in TCM for PD neuroinflammation due to oxidative stress

As illustrated in Table 4.

**Table 4:** Active ingredients in TCM for PD neuroinflammation due to oxidative stress.

Name	Experimental PD Models	Effects on inflammation	Effects on cytokine levels	Reference
Baicalin	MPTP-induced mice	Nrf2/HO-1↑, Microglial activation, NF-κB↓	Keap1, Nrf2, HO-1↑, Iba-1, NLRP3, TNF-α, IL-1β, IL-6↓	^[190]^
	LPS-induced mice	Nrf2/HO-1↑	Nrf2, HO-1↑, IL-1β, TNF-α↓	^[191]^
Icariin	6-OHDA-induced mice	Nrf2/HO-1↑, Microglial activation, Astrocytic activation↓	Keap1, HO-1, NQO1↑, Iba-1, GFAP, TNF-α, iNOS↓	^[192]^
Isoliquiritigenin	6-OHDA-induced mice	Nrf2/NQO1↑, Microglial activation↓	Iba-1, IL-1β, IL-6, TNF-α↓	^[193]^
Apigenin	LPS-induced rats	Nrf2/HO-1↑, TLR4/NF-κB↓	TNF-α, IL-1β, IL-6, iNOS, NO↓	^[194]^
Luteolin	BV2 cells from rotenone-induced rats	Nrf2↑	IL-1β↓	^[195]^

PD = Parkinson disease, TCM = traditional Chinese medicine.

#### 3.4.1. Baicalin

Baicalin emerges from the revered Chinese herb *Scutellaria baicalensis* as a flavonoid of notable potency. Its role in MPTP-induced PD mice models is pivotal, catalyzing the Nrf2/HO-1 pathway’s activation, which escalates Keap1, Nrf2, and HO-1 levels, curtailing oxidative stress. Moreover, its ability to downregulate Iba-1, stifle microglial activation, and dampen the NLRP3 inflammasome’s zeal by suppressing NF-κB, effectively diminishes TNF-α, IL-1β, and IL-6 levels, offering solace from neuroinflammation.^[190]^ Baicalin prowess extends to LPS-induced PD mice, where it elevates Nrf2 and HO-1, curbing IL-1β and TNF-α secretion and further easing neuroinflammation.^[191]^

#### 3.4.2. Icariin

In the milieu of 6-OHDA PD models, Icariin champions the Nrf2/HO-1 pathway, enriching the release of Keap1, HO-1, and NQO1, thus shielding against oxidative stress. Its suppression of GFAP and Iba-1 expression, alongside the inhibition of microglia and astrocytes, trims TNF-α and iNOS levels, mitigating neuroinflammation and safeguarding dopaminergic neurons.^[192]^

#### 3.4.3. Isoliquiritigenin

In the context of 6-OHDA PD mouse, isoliquiritigenin marks its territory by downregulating Iba-1, impeding microglial activation. Through the Nrf2/NQO1 pathway, it diminishes IL-1β, IL-6, and TNF-α levels, stanching the flow of neuroinflammation.^[193]^

#### 3.4.4. Apigenin

Apigenin, within LPS-induced PD rats, reduces striatal concentrations of TNF-α, IL-1β, IL-6, iNOS, and NO by downregulating the TLR4/NF-κB pathway with precision and simultaneously amplifying the Nrf2/HO-1 pathway’s voice, thus dialing down neuroinflammation.^[194]^

#### 3.4.5. Luteolin

In BV2 microglial cells from rotenone PD models, luteolin ascends by upregulating the Nrf2 pathway, clamping down on microglial activation. This strategic maneuver decreases IL-1β levels, quelling the storm of neuroinflammation.^[195]^

### 3.5. Active ingredients in TCM for PD inflammation through the GBA

As illustrated in Table 5.

**Table 5:** Active ingredients in TCM for PD inflammation through the GBA.

Name	Experimental PD models	Effects on inflammation	Effects on cytokine levels	Reference
Curcumin	MPTP-induced mice	SIRT1/NRF2, Intestinal cell pyroptosis↓, Protect the intestinal barrier	Intestinal IL-1β, IL-6, IL-18, TNF-α↓	^[196,197]^
Resveratrol	MPTP-induced mice	Intestinal inflammation↓, Protect the intestinal barrier	Intestinal TNF-α, IL-6, IL-1β↓	^[198,199]^
FLZ	Rotenone-induced mice	TLR4/MyD88/NF-κB, Intestinal inflammation↓, Protect the BBB	Intestinal IL-1β, IL-6, TNF-α, iNOS, COX-2↓	^[200]^
KRG	MPTP-induced mice	Intestinal inflammation, Tight junction proteins↓, Protect the intestinal barrier	Intestinal TNF-α, IL-1β, α-synuclein↓	^[201]^
Diosgenin	MPTP-induced mice	Bile acid, GLP-1↑, NF-κB, CNS Microglial activation, Astrocytic activation↓	Intestinal IL-6, NOX2, ICAM, CNS GFAP, Iba-1↓	^[202–205]^
CPS	MPTP-induced mice	DAO, S-IGA↑, Emr1, Microglial activation↓, Protect the intestinal barrier	TNF-α, IL-β, IL-6↓	^[206]^
Morin	Rotenone-induced mice	Intestinal inflammation, CNS Microglial activation, Astrocytic activation↓, Protect the intestinal barrier	Iba-1, GFAP, TLR4↓	^[207]^

GBA = gut-brain axis, PD = Parkinson disease, TCM = traditional Chinese medicine.

#### 3.5.1. Curcumin

In the challenging environment of MPTP-induced PD mouse models, curcumin shines as a beacon of hope. Through the SIRT1/NRF2 pathway, it significantly lowers the concentrations of IL-1β, IL-6, IL-18, and TNF-α in the gut, combats intestinal cell pyroptosis-induced inflammation, and fosters a healthier gut microbiome. This sequence of actions fortifies the intestinal barrier, reduces transmissions along the GBA, and notably diminishes neuroinflammation. The culmination of these effects is the protection of vital dopaminergic neurons and the amelioration of motor dysfunctions in affected mice.^[196,197]^

#### 3.5.2. Resveratrol

In MPTP PD mouse, resveratrol emerges as a powerful ally for the gut microbiome, enhancing its potential and bolstering the intestinal barrier’s functionality.^[198]^ Remarkably, through the innovative approach of fecal microbiota transplantation, it corrects the imbalance within the gut microbiome, leading to a reduction in intestinal TNF-α, IL-6, and IL-1β levels. This strategy effectively curtails both intestinal and neuroinflammation through the GBA, offering a shield for dopaminergic neurons and mitigating motor dysfunctions.^[199]^

#### 3.5.3. FLZ

FLZ demonstrates its efficacy in rotenone-induced PD mice by rejuvenating the gut microbiome, alleviating intestinal dysfunction, and curtailing inflammation and permeability through the TLR4/MyD88/NF-κB pathway. It lowers the levels of IL-1β, IL-6, TNF-α, iNOS, and COX-2, thereby enhancing the BBB’s integrity. By moderating neuroinflammation via the gut-brain axis, it safeguards dopaminergic neurons, rectifies the irregular gait, and boosts walking speed in PD mice.^[200]^

#### 3.5.4. Korean Red Ginseng

Korean Red Ginseng, rich in ginsenosides, in MPTP PD mice, plays a critical role by decreasing intestinal TNF-α and IL-1β levels, soothing intestinal inflammation, and promoting the expression of tight junction proteins. This not only protects the intestinal barrier and lowers permeability but also guards intestinal dopaminergic neurons and curtails intestinal α-synuclein levels. Through the gut-brain axis, it extends its protective arm to SNpc dopaminergic neurons, thus repressing abnormal rotational behaviors.^[201]^

#### 3.5.5. Diosgenin

Diosgenin is a steroidal saponin extracted from Dioscorea.^[202]^ Diosgenin engages with the gut microbiome to regulate the synthesis of bile acids from cholesterol, which, in turn, influences the metabolic landscape of the gut microbiome.^[203,204]^ In MPTP-induced PD mice, diosgenin amplifies bile acid expression and, via the GLP-1 pathway mediated by bile acids, suppresses the intestinal NF-κB pathway. This leads to decreased IL-6, NOX2, and ICAM levels, reducing intestinal inflammation and, by extension, neuroinflammation via the gut-brain axis. This intricate interplay results in the protection of dopaminergic neurons and improvements in gait irregularities and walking speed.^[205]^

#### 3.5.6. Polysaccharides from *Chlorella pyrenoidosa* (CPS)

In the context of the MPTP PD mouse, CPS enhances DAO and S-IGA levels, which are crucial for maintaining intestinal barrier integrity. By downregulating Emr1 expression via the GBA and inhibiting microglial activation, they effectively reduce TNF-α, IL-β, and IL-6 levels, suppressing neuroinflammation and protecting dopaminergic neurons, thus alleviating motor slowness.^[206]^

#### 3.5.7. Morin

Morin offers a multifaceted approach to combatting PD in rotenone-induced models. By alleviating colon mucosal epithelial cell necrosis, it preserves the intestinal barrier and reduces inflammation. Furthermore, it downregulates Iba-1 and GFAP expression, inhibits SNpc microglia and astrocyte activation, and mitigates neuroinflammation by suppressing the TLR4 signaling pathway. This comprehensive action protects dopaminergic neurons and ameliorates motor coordination and dysfunction.^[207]^

### 3.6. Potential side effects and clinical feasibility of TCM ingredients

The therapeutic strategies discussed provide foundational evidence for managing inflammation in PD and may emerge as viable treatment options. Active components such as hyperoside and astragaloside IV can modulate pathways like NF-κB, NLRP3, and JAK/STAT, thus inhibiting the activation of neuroglial cells and reducing neuroinflammation in PD.^[124,158]^ Similarly, compounds like baicalin and icariin act on the Nrf2/HO-1 signaling pathway, diminishing neuroglial cell activation and thereby alleviating oxidative stress-induced neuroinflammation.^[190–192]^ Other agents like curcumin and FLZ inhibit the SIRT1/NRF2 and TLR/NF-κB pathways while also safeguarding the gut and blood-brain barriers, thus mitigating peripheral inflammation triggered via the GBA.^[196,197,200]^ These mechanisms unlock new potentials for treating PD inflammation.

Although TCM ingredients generally exhibit a high safety profile, they are not devoid of risks and side effects. Despite numerous studies highlighting berberine’s benefits for PD, its potential to enhance the neurotoxicity of 6-OHDA and attenuate the pharmacological efficacy of levodopa warrants attention.^[208]^ Ongoing investigations into berberine’s interactions with levodopa are vital to minimize clinical risks. Glycyrrhizin’s principal adverse effect involves altering adrenal cortical hormone metabolism, leading to hypertension and hypokalemia. In PD, glycyrrhizin might disrupt the effects of dopaminergic agonists due to its impact on mineralocorticoid receptors, necessitating cautious application.^[209]^ These side effects are manageable with targeted antihypertensive medications and potassium supplements, alongside regular monitoring of blood pressure and potassium levels to mitigate potential risks. Triptolide is notorious for its toxicity and can induce liver and kidney dysfunction, including hepatocellular injury, hepatitis, renal failure, or nephritis. Given that the liver and kidneys are pivotal in drug metabolism, dose management and regular monitoring of these organ functions are imperative. Immediate drug cessation and symptomatic treatment are required if liver or kidney impairment occurs due to triptolide.^[210]^ Resveratrol may trigger gastrointestinal issues such as nausea and diarrhea, and increase liver enzyme levels (ALT and AST), potentially harming the liver.^[211]^ While current studies scarcely address the specific interactions between resveratrol and PD medications, resveratrol’s influence on liver metabolic enzymes, particularly the CYP450 family, could alter the metabolism of PD drugs. Avoiding the concurrent use of resveratrol with drugs metabolized by hepatic enzymes is advisable to prevent potential interactions. Future research should focus on circumventing these risks and side effects, a critical step toward the broad clinical deployment of these medicinal compounds. Additionally, adjusting drug dosages according to individual patient variables such as age, weight, and liver or kidney function, and employing combinatory drug regimens with regular patient monitoring, could further reduce adverse effects.

## 4. Discussion: unraveling the role of inflammation in PD

Inflammation emerges as a pivotal player in the pathogenesis of PD, not merely as an outcome but as a persistent undercurrent driving the disease forward. This study delves into the intricate relationship between inflammation and the neurodegenerative trajectory of PD, spotlighting the efficacy of TCM components in curtailing PD-associated inflammation. Our findings illuminate a direct link between heightened inflammatory markers and neuronal damage in PD patients, reinforcing the critical role of inflammation in the disease’s evolution.

Echoing prior studies, we noted a marked increase in microglial and astrocytic activation within the brains of PD patients – a hallmark of CNS inflammation.^[14,15,17–19,41,45,47]^ This activation is orchestrated via pathways such as TLR4/NF-κB/NLRP3, p38/MAPK, and JAK2/STAT3,^[36]^ catalyzing neuroinflammation. Notably, TCM compounds, including Quercetin^[124,125]^ and Resveratrol,^[127,128]^ have shown promise in inhibiting this cellular activation and diminishing inflammatory mediator levels, thereby mitigating neuroinflammation. Moreover, oxidative stress, a known promoter of neuroinflammation, is counteracted by TCM compounds like Icariin^[192]^ and Isoliquiritigenin^[193]^ through the Nrf2/HO-1 pathway. Additionally, peripheral inflammation, facilitated through the gut-brain axis, is addressed by TCM compounds such as Curcumin^[196,197]^ and Diosgenin,^[205]^ highlighting a multifaceted approach to suppressing neuroinflammation. Elevated levels of inflammatory cytokines in PD patients underscore the significance of these mediators in neurodegenerative processes, aligning with existing research and underscoring the pivotal role of inflammation in PD progression.^[37–39]^

However, our study, while in agreement with the current literature, acknowledges certain limitations. Notably, the role of autoimmune inflammation centered around SNCA,^[118]^ involving B cells, T cells, NK cells, and microglia, among others,^[212]^ and the impact of TCM components on this aspect remain underexplored, pointing towards fertile ground for future investigations. Additionally, while the therapeutic effects of TCM active ingredients such as Polyphenols,^[213]^ Flavonoids,^[214]^ Saponins,^[215]^ and Terpenes^[216]^ on PD models have achieved some success, their translation from theory to clinical practice in PD treatment awaits realization. TCM, with its rich repository of medicinal herbs, presents untapped potential in uncovering novel mechanisms of action against PD, urging further exploration of both lesser-known herbs and diverse compounds within established medicinal plants: (1) Develop TCMs with currently known fewer effective ingredients, such as *Uncaria rhynchophylla*, Hematite, and Tortoise shell; (2) Continue to explore different active ingredients in existing TCM, such as Ginseng, Astragalus, White Peony, and Rehmannia.

## 5. Conclusion and future perspectives: harnessing TCM for PD

TCM components offer a beacon of hope in suppressing neuroinflammation and peripheral inflammation in PD, through targeted inhibition of pathways like TLR4/NF-κB, p38/MAPK, JAK2/STAT3, and bolstering of the Nrf2/HO-1 pathway. This approach promises a reduction in inflammatory mediators such as TNF-α, IL-1β, IL-6, iNOS, and COX-2,^[37–39]^ paving the way for alleviating neuroinflammation and potentially halting the progression of PD. Despite the current absence of a cure, the quest for novel therapeutic strategies remains undeterred, with advancements in drug development, identification of new pathways and targets, and the exploration of stem cell therapies^[217]^ and gene therapy^[218]^ offering promising avenues for future treatment options. Our collective hope is for a future where the burden of PD is significantly reduced, improving the quality of life for those affected by this debilitating condition.

## Author contributions

**Conceptualization:** Yuxuan He.

**Data curation:** Jingyi Wang.

**Formal analysis:** Chunmiao Ying.

**Funding acquisition:** Yunke Zhang.

**Project administration:** Yunke Zhang.

**Software:** Weixiao Liang.

**Supervision:** Boqiao Wang.

**Validation:** Zaitian Yin.

**Visualization:** Jing Gao.

**Writing – original draft:** Yuxuan He.

**Writing – review & editing:** Xiaohui Zhao.
